# Key Limitations and New Insights Into the *Toxoplasma gondii* Parasite Stage Switching for Future Vaccine Development in Human, Livestock, and Cats

**DOI:** 10.3389/fcimb.2020.607198

**Published:** 2020-11-25

**Authors:** Marie-Noëlle Mévélec, Zineb Lakhrif, Isabelle Dimier-Poisson

**Affiliations:** Team BioMAP, Université de Tours, INRAE, ISP, Tours, France

**Keywords:** *T. gondii*, tachyzoite, brabyzoite, sporozoite, differentiation, immunity, vaccine

## Abstract

Toxoplasmosis is a parasitic disease affecting human, livestock and cat. Prophylactic strategies would be ideal to prevent infection. In a One Health vaccination approach, the objectives would be the prevention of congenital disease in both women and livestock, prevention/reduction of *T. gondii* tissue cysts in food-producing animals; and oocyst shedding in cats. Over the last few years, an explosion of strategies for vaccine development, especially due to the development of genetic-engineering technologies has emerged. The field of vaccinology has been exploring safer vaccines by the generation of recombinant immunogenic proteins, naked DNA vaccines, and viral/bacterial recombinants vectors. These strategies based on single- or few antigens, are less efficacious than recombinant live-attenuated, mostly tachyzoite *T. gondii* vaccine candidates. Reflections on the development of an anti-*Toxoplasma* vaccine must focus not only on the appropriate route of administration, capable of inducing efficient immune response, but also on the choice of the antigen (s) of interest and the associated delivery systems. To answer these questions, the choice of the animal model is essential. If mice helped in understanding the protection mechanisms, the data obtained cannot be directly transposed to humans, livestock and cats. Moreover, effectiveness vaccines should elicit strong and protective humoral and cellular immune responses at both local and systemic levels against the different stages of the parasite. Finally, challenge protocols should use the oral route, major natural route of infection, either by feeding tissue cysts or oocysts from different *T. gondii* strains. Effective *Toxoplasma* vaccines depend on our understanding of the (1) protective host immune response during *T. gondii* invasion and infection in the different hosts, (2) manipulation and modulation of host immune response to ensure survival of the parasites able to evade and subvert host immunity, (3) molecular mechanisms that define specific stage development. This review presents an overview of the key limitations for the development of an effective vaccine and highlights the contributions made by recent studies on the mechanisms behind stage switching to offer interesting perspectives for vaccine development.

## Introduction

*Toxoplasma gondii* (*T. gondii*) is an obligate intracellular protozoan parasite that infects humans, domesticated and wild warm-blooded animals. As a result, the parasite has a world-wide distribution (Dubey, 2008). *Toxoplasma* infection is acquired by consumption of oocysts shed from cats in contaminated water or vegetables or by ingestion of tissue cysts contained in infected meat. *T. gondii* can undergo both asexual and sexual replications in cats and members of the feline family (definitive hosts), but can divide only asexually in all other warm-blooded mammals including humans (intermediate hosts). Tachyzoites, bradyzoites contained in tissue cysts and sporozoites contained in sporulated oocysts are the three infectious stages of *T. gondii.*

After ingestion of *T. gondii* oocysts or cysts by an intermediate host, the sporozoites or bradyzoites released into the lumen of the small intestine, pass through the intestinal epithelial barrier and rapidly undergo multiplication by endodyogeny within the parasitophorous vacuole (PV) inside various cell types. Tachyzoites disseminate throughout the organism after infecting circulating cells such as dendritic cells, natural killers, monocytes and macrophages (Courret et al., 2006; Persson et al., 2009). In addition, tachyzoites are capable of crossing the placental blood barrier to infect the fetus. Then, under the pressure of the host immune system, tachyzoites transform into bradyzoites, the slow replicating form of parasite. The encysted bradyzoites persist inside the host which correspond to establishment of chronic infection and are found in a variety of tissues including heart, skeletal muscle, lung and brain (Remington and Cavanaugh, 1965; Di Cristina et al., 2008). Upon immune suppression, bradyzoites will transform back into proliferating tachyzoites. In definitive host, bradyzoites invade the intestinal epithelium and differentiate into five morphologically distinct types of schizonts designated A through E (Dubey and Frenkel, 1972). Type E schizonts give rise to merozoites which differentiate into gametes. Males (microgametes) fertilize females (macrogametes) to produce diploid oocysts which develop thick impermeable walls and are shed in the feces (Tenter et al., 2000). Once sporulation occurs (1 to 5 days following secretion), oocysts are infectious for an extended period of time, depending on environmental conditions.

Felids acquire infection by carnivorism by ingesting prey tissue containing cysts or, more rarely, oocysts. From a systematic review and meta-analysis (from 1967 to 2017) the worldwide seroprevalence of *T. gondii* has been estimated to be 35% in domestic cat and 51% in wild felids respectively (Montazeri et al., 2020). *T. gondii* infections are highly prevalent in both sheep and goats and have been found in small ruminants worldwide (Stelzer et al., 2019). For example, in Europe seroprevalence values ranging from 24.5% to 89% have been reported in sheep (Stelzer et al., 2019). Ingestion of oocysts through contaminated fodder or water is the most important route of infection in small ruminants. Seroprevalences in pig varied according to management system in particular outdoor access is an important risk factor of infection, age, pig categories and geographic areas (Stelzer et al., 2019). Lower prevalence (<1%) is observed in pigs reared in indoor farms with control management conditions, whereas higher prevalence values (>60%) are found in farms without controlled conditions allowing outdoor access (De Berardinis et al., 2017). Most *T. gondii* infections in pigs are acquired by the ingestion of food/water contaminated with oocysts or by ingestion of intermediate hosts harboring tissue cysts (Stelzer et al., 2019). *T. gondii* is estimated to infect one-third of the human population with prevalence varying from 10% to over 50% according to geographic areas (Robert-Gangneux and Dardé, 2012). As omnivorous, humans are exposed to both *T. gondii* tissue cysts and oocysts in their diet through consumption of undercooked meat and water or vegetables contaminated with cysts or oocysts respectively. Isolation of viable parasites from tissues of pig and sheep confirm that these species represent a risk for human transmission (Halos et al., 2010; Alves et al., 2019; Miura et al., 2019). Recently, serologic tests able to distinguish oocyst- versus meat- induced infections, revealed that environmental oocyst transmission is a more important source of transmission than previously thought (Boyer et al., 2011; Hill et al., 2011).

In cat, *T. gondii* infection is generally asymptomatic. The host immunity is able to limit oocyst shedding to a short period usually 1–3 weeks (Dubey and Frenkel, 1974) and to protect against re-shedding after re-infection with homologous strain (Davis and Dubey, 1995; Dubey, 1995). However, partial or no protection against re-shedding is observed after second infection with heterologous strains (Zulpo et al., 2018). In sheep, acute toxoplasmosis is characterized by a brief episode of fever and lack of appetite (Stelzer et al., 2019). A primary maternal infection is a leading cause of stillbirth and preterm lamb loss. In Europe and USA, 10 to 23% of ovine abortions are caused by *T. gondii* (Dubey, 2009). In most cases, *T. gondii* infection in pig is subclinical, nevertheless, clinical disease in young animals and reproductive failure in sows have been reported worldwide (Stelzer et al., 2019). In healthy humans, primary infection with *T. gondii* is usually asymptomatic, nevertheless, flu-like symptoms can occur. *T. gondii* infections are more detrimental in immunocompromised individuals, such as those with HIV infection, patients receiving organ transplants or undergoing cancer treatment where primary infection or reactivation of latent tissues cysts results in encephalitis and pulmonary toxoplasmosis. A primary infection during pregnancy may result in congenital infection causing abortion or fetal abnormalities, including hydrocephalus, intracranial calcifications and retinochoroiditis (Montoya and Liesenfeld, 2004). In the last years, ocular toxoplasmosis was identified not only following congenital infection but also in cases of postnatally acquired infections (Smith et al., 2020; Zhao and Ewald, 2020).

*T. gondii* disease severity is determined by many factors, including the host species and for each species the host genetic background, the genotype of the parasite and the stage that is acquired by the host, oocysts being more virulent than bradyzoites in tissue cysts (Lindsay and Dubey, 2020). It is now apparent that many atypical *T. gondii* genotypes exist besides the typical 3 genotypes (type I, type II and type III) first described in Europe and United States. In Europe, type II strains and type III strains are dominant, while types II, III, and 12 are dominant in North America. In Asia and Africa, (types II and III) and regional clonal lineages such as Chinese 1 in China or Africa 1 and Africa 3 in Africa are dominant. In contrast, the genetic diversity is much higher in South America, with no clear dominance of any particular genotype (Galal et al., 2019). An association between the *T. gondii* strains identified in South America and the incidence of severe ocular and systemic toxoplasmosis in immunocompetent patients has been made (Holland, 2003; Grigg et al., 2015).

Currently, drug therapies are ineffective against *T. gondii* cysts in tissues (Dunay et al., 2018). So, prophylactic strategies would be ideal to prevent infection. The observation that *T. gondii* induces a protective immunity in most hosts (animal species including people) suggest that an immunoprophylactic strategy is a realistic goal. In a One Health vaccination approach the objectives would be the prevention of congenital disease in both women and livestock species such as sheep and goats; prevention/reduction of *T. gondii* tissue cysts in food-producing animals; and prevention/reduction oocyst shedding in cats (Innes et al., 2019). Over the past 30 years, many vaccine candidates have been tested, mostly in inbred mice, using various strategies (reviewed in Faridnia et al., 2018; Dodangeh et al., 2019; Loh et al., 2019; Rezaei et al., 2019; Wang et al., 2019; Pagheh et al., 2020). Varying degrees of protective immunity have been achieved with most antigens tested. However, as the genetic background of mice influence the outcome of the vaccine trial, and as there is no standardized challenge protocol, comparison is difficult. Although promising, these strategies based on single or few antigens, are less efficacious than recombinant live-attenuated, mostly tachyzoite *T. gondii* vaccine candidates (**Table 1**).

**Table 1:** *T. gondii*, RH, PRU, ME49 attenuated live vaccines evaluated in mouse model.

Targeted gene	Immunizationmouse/amount/route	Challenge*T. gondii^1^*/amount/route	Protection Acute^2^ Chronic^3^		References
**RH**	**Tachyzoites**	**Type I**			
Carbamoyl phosphate synthetase II (CPSII)	BALB/c, 10^5^, i.p.	RH I, 200 T, i.p.	100%		Fox and Bzik, 2002
	C57BL/6, 10^6^, i.p	ME49 II, 100 C, oral	100%		Gigley et al., 2009
		ME49 II, 10 C, oral		99-93%	
MIC1+ MIC3	Swiss OF1, 20, i.p.	76K II, 45 C, oral		>96%	Cérède et al., 2005
					Ismael et al., 2006
MIC2	BALB/c, 5x10^4^, i.p.	RH I, 150 T, i.p.	100%		Huynh and Carruthers 2006
Ribosomal protein (RPS13)	Swiss OF1, 10^5^, i.p.	RH I, 2x10^3^ T, i.p.	100%		Hutson et al., 2010
		ME49 II, 50 C, i.p.		94%	
Ortidine monophosphate decarboxylase (OMPDC)	C57BL/6, 10^6^, i.p.	RH I, 200 T, i.p.	100%		Fox and Bzik, 2010
OMPDC + Uridine phosphorylase (UP)	C57BL/6, 10^6^, i.p.	RH I, 200 T, i.p.	100%		Fox and Bzik, 2010
Apical membrane antigen 1 (AMA1)	BALB/c, 10^5^, i.p.	RH I, 10^5^ T, i.p.	100%		Lagal et al., 2015
	C57BL/6J, 250, i.p.	RH I, 10^3^ T, i.p.	100%		
	CD-1, 10^4^, i.p.	RH I, 10^5^ T, i.p.	100%		
	BALB/C, 10^5^, i.p.	ME49 II, 10^3^ T, i.p.		70%	
Phosphatidyl threonine synthase (PTS)	C57BL/6J, 500, i.p.	RH I, 50 T, i.p.	100%		Arroyo-Olarte et al., 2015
		ME49 II, 3 C, i.p.		ND^4^	
GRA17	Kunming, 5x10^4^, i.p.	PRU II, 20 C, oral		98%	Wang et al., 2017
		RH I, 10^3^ T, i.p.	100%		
Novel Putative Transporter 1 (NPT1)	Kunming, 10^6^, i.p.	RH I, 10^3^ T, i.p.	100%		Yang et al., 2019
		PYS 9, 10^3^ T, i.p.	100%		
		PRU II, 20 C, oral		98%	
GRA17+NTP1	Kunming, 10^6^, i.p.	RH I, 10^3^ T, i.p.	100%		Liang et al., 2020
		PYS 9, 10^3^ T, i.p.	100%		
		PRU II, 100 C, oral	100%		
		PRU II, 100 O, oral	100%		
Tyrosine kinase–like 1 (TKL1)	Kunming, 10^6^, i.p.	RH I, 10^3^ T, i.p.	100%		Wang J. L. et al., 2020
		PYS 9, 10^3^ T, i.p.	100%		
		PRU II, 100 C, oral	100%		
		PRU II, 100 O, oral	100%		
**PRU**	**Tachyzoites**	**Type II**			
OMPDC	C57BL/6, 3x10^6^, i.p.	RH I, 10^3^ T, i.p.	100%		Fox and Bzik, 2015
		PRU II, 2x10^7^ T, i.p.		100%	
Lactate dehydrogenase (LDH1, 2)	BALB/c, 10^3^, i.p	RH I, 100 T, i.p.	100%		Abdelbaset et al., 2017
Ca2+-dependent protein Kinase 2	Kunming, 500, i.p	RH I, 10^3^ T, i.p.	100%		Wang J. L. et al., 2018
		PYS 9, 10^3^ T, i.p.	100%		
		PRU II, 20 C, oral		98%	
GRA17	Kunming,, 10^6^, i.p.	RH I, 10^3^ T, i.p.	100%		Li et al., 2020
		PYS 9, 10^3^ T, i.p.	100%		
		PRU II, 100 C, oral	100%		
		PRU II, 10 C, oral		98%	
**ME49**	**Tachyzoites**	**Type II**			
LDH1+ LDH2	ICR, 10^4^, i.p.	ME49 II, 10^4^ T, i.p.	100%		Xia et al., 2018
		VEG III, 10^4^ T, i.p.	100%		
		C7719 Chinese 1,10^4^ T, i.p.	100%		
OMPDC	ICR, 10^4^, i.p	ME49 II, 10^4^ T, i.p.	100%		Xia et al., 2018
Adenylosuccinate lyase (ADSL)	ICR, 100, i.p.	RH I, 500 T, i.p.	100%		Wang L. et al., 2020
		ME49 II, 10^3^ T, i.p.	100%		

i.p., intraperitoneal route; T, tachyzoite; C, cyst; O, oocyst.

^1^challenge: T. gondii strain and Type/genotype.

^2^protection against acute toxoplasmosis: non-vaccinated control groups : 100% mortality, except for Wang L. et al. (2020) where 80% mortality, was recorded following challenge with ME49 tachyzoites.

% survival monitored more than 30 days post-challenge.

^3^protection against chronic toxoplasmosis : % brain tissue cyst reduction/nonvaccinated control group.

^4^brain cysts were not detected (ND).

New approaches to develop effective vaccines are needed and depend on a better understanding of host-parasite interactions and parasite biology. This review presents the progress on our knowledge of the (1) protective host immune response during *T. gondii* invasion and infection in the different hosts, (2) manipulation and modulation of host immune response to ensure survival of the parasites able to evade and subvert host immunity, (3) molecular mechanisms that define stages specific development. This review concentrates, where possible, on studies using the natural oral route of infection, either by feeding tissue cysts or oocysts of the relevant host.

## Immunity Against *T. gondii*: The Host Perspective

Except in the congenital cases, natural infection occurs through the oral route after ingestion of tissue cysts or oocysts. Bradyzoites or sporozoites released from cysts or oocysts respectively invade intestinal cells and convert to tachyzoites, the fast multiplying stage leading to the rapid spread of the parasite throughout the whole body. Tachyzoites are not completely eliminated by the immune system, they differentiate into bradyzoites and persist as bradyzoite-containing cysts. In cats, both sexual and asexual reproductive stages occur after ingestion of tissue cysts or oocysts. The sexual cycle is restricted to the feline intestine. As a result, male and female gametes are formed and after fertilization immature oocysts are created and excreted into the environment *via* feces.

As immediate reaction to host cell invasion by the parasite, an innate immune response is activated followed by an adaptive immune response resulting in antigen presentation and activation of the antigen specific T and B cell response. In most cases the immune response is capable of controlling the acute phase of the infection but is unable to eradicate the tissue cysts and is protective against reinfection. A wealth of information has been obtained in murine model to study immune responses against *T. gondii*. All data acquired in mouse cannot be directly transposed to humans, cats and live stocks. However, due to the intracellular nature of the parasite, protective immunity to *T. gondii* is recognized to be primarily dependent on the cellular immunity mediated by both CD4^+^ and CD8^+^ T cells, interferon-gamma (IFN-γ) being identified as a key mediator of protection against both acute and chronic *T. gondii* infection in mice (Suzuki and Remington, 1988; Gazzinelli et al., 1991; Casciotti et al., 2002). *T. gondii* infection also promotes antibody responses in systemic and mucosal compartments. Although their action is limited to only extracellular parasites, antibodies can opsonize parasites for phagocytosis, block parasite cell invasion, and also activate the classical complement pathway. An interesting aspect is the specific IgA response in the gut mucosa that thought to be an important barrier to oral re-infection.

### Cat Immunity

In the intestine of cat, 96 h post-oral infection with *T. gondii* cysts, upregulation of class I major histocompatibility complex (MHC) I related genes suggest that cats promote potent antigen-specific immunity to limit replication of the parasite (Wang M. et al., 2018). At the systemic level, peripheral blood mononuclear cells (PBMCs) isolated from blood cat samples produced IFN-γ as early as 4 days post-oral infection with *T. gondii* cysts when stimulated with *T. gondii* antigens (Yin et al., 2015). It has also been shown that IFN-γ and interleukin-12 (IL-12) genes are upregulated in cat mesenteric lymph nodes (MLNs) and spleen 7 days following oral infection (Koyama et al., 1999). More recently, RNA-sequencing was used to detect transcriptional changes, 7 days after cat oral infection with *T. gondii* cysts, in different cat tissues (small intestine, lung, heart, liver, spleen, brain). In almost all tissues, cytokine-cytokine receptor interaction, Jak-STAT signaling pathway, NOD-like receptor signaling pathway, NF-κB signaling pathway, MAPK signaling pathway, T cell receptor signaling pathway and the cytosolic DNA sensing pathway, were among the up-regulated immune pathways. Among co-expressed genes, IFN-γ induced Indoleamine 2,3-dioxygenase (IDO) was co-expressed in tissues: heart, liver, lung, small intestine, and spleen, but not in the brain tissue. IDO enzyme degrades L-tryptophan an amino acid for which *T. gondii* is auxotrophic. IFN-γ inducible Guanylate-binding protein (GBP) was detected in the heart, liver, lung, and spleen, but not in small intestine and brain. GBP is a member of the GTPase family. GBPs accumulate on PVs of *T. gondii* to mediate cell autonomous immunity (Cong et al., 2018). After challenge *via* the carotid artery with *T. gondii* tachyzoites, precapsular or popliteal lymph nodes cytokine mRNA levels were quantitated by reverse transcription (RT)-quantitative competitive polymerase chain reaction (PCR) (Levy et al., 2004). Increased expression of IL-12, tumor necrosis factor alpha (TNF-α), IFN-γ, and IL-6 that peaked at 7 days post-challenge was observed. IL-2 expression increased earlier, at 3 days post-infection. An inflammatory response was found in naturally *T. gondii* infected cats, asymptomatic for toxoplasmosis (Faria et al., 2018). Serum levels of TNF-α, reactive oxygen species (ROS), and nitric oxide (NO) were higher in seropositive cats compared to seronegative cats.

*T. gondii* oral infection promotes antibody responses. Anti-*T. gondii* IgM and IgG antibodies were detected 10 and 14 days post-infection (Dubey et al., 1995). Serum IgA were detected later, on week 34 after infection (Burney et al., 1995). Anti-T*. gondii* IgA antibodies were also detected in the intestinal tract of *T. gondii* infected cats (Rush et al., 2001). IgA in aqueous humor were also detectable in some cats (Lappin et al., 1995).

### Sheep Immunity

The few studies carried out to investigate the cellular immune response in *T. gondii* infected sheep, found that the early immune responses involved IFN-γ. Within 2 to 5 days post-infection IFN-γ was detected and persisted for 6 to 9 days in the efferent duct of a lymph node draining the site of subcutaneous inoculation with *T. gondii* tachyzoites (Innes et al., 1995a). T cells were the major population present in responding efferent lymph, the CD4^+^ T cells initially being the predominant subpopulation. Days 9–10 after infection, CD8^+^ T cells were the majority subpopulation. Efferent lymph cells produced IFN-γ when stimulated* in vitro* with *T. gondii *antigens from day 6 after infection (Innes et al., 1995b). Simultaneous IFN-γ and IL-12 responses were produced by *in vitro* stimulated mesenteric lymphocytes and splenocytes from sheep infected 4 days earlier with *T. gondii* cysts by the oral route (Verhelst et al., 2014). In PBMCs directly isolated from sheep infected orally with *T. gondii* cysts, a clear increase in IFN-γ mRNA expression, determined by quantitative RT-PCR, was observed 2 weeks post-infection which remained until 7 weeks post-infection, the end of the observation period. In contrast, IL-10 and IL-4 mRNA expression did not show a consistent increase in all animals (Verhelst et al., 2015). Similarly, IFN-γ production was found in supernatants from PBMCs stimulated with *T. gondii* antigens, collected from sheep infected orally with *T. gondii* oocysts, 2 weeks post-infection (Stanley et al., 2004).

Recently, the peripheral and placental immune responses in pregnant sheep after oral infection with *T. gondii* oocyst at different times of gestation, have been studied (Castaño et al., 2019). An early peripheral release of IFN-γ at the first week post-infection followed by a short peak of IL-10 and TNF-α at the second week post-infection was observed in maternal sera, without significant differences between infection at the first, second and last term of gestation, with the exception of TNF-α, which was higher on those animals infected at mid-gestation. Studying the cytokine transcript expression profile at the maternofetal interface, a mixed of T Helper 1 (Th1) and Th2 type placental immune response was detected. IFN-γ showed the highest fold increase after infection at the first, second and last term of gestation. IL-4 showed higher levels on the first and second terms, while IL-10 showed a clear increase at the second and third terms of gestation.

In addition to cellular mechanisms, *T. gondii* infection in sheep stimulates humoral immune responses. Sheep orally infected with oocysts mount an IgG response by 2 weeks post-infection. Maximum serologic titers are detected from 21 to 56 days post-infection (Tenter et al., 1992; Stanley et al., 2004; Lopes et al., 2009; Dos Santos et al., 2016). *T. gondii* specific IgM antibodies peaked at 3 weeks post-infection and preceded an IgG response (Trees et al., 1989).

### Pig Immunity

Following oral infection of pig with *T. gondii* oocysts, a significant increase in IFN-γ, IL-15, TNF-α, and inductible-NO gene expressions, in lymph nodes draining sites of infection colonic lymph nodes (CLNs), jejunal Peyer’s patches (PPs) and MLNs, was observed at 7 days post-infection. Commensurate with recovery from overt disease, the proinflammatory gene expression was reduced at day 14 post-infection. Along with the Th1-like pattern of gene expression an increased anti-inflammatory gene expression is also observed. The IL-10 gene expression is significantly increased at 7 days post infection in MLNs and CLNs. The expression of transforming growth factor-β significantly increased in the CLNs at 7 post-infection and increased in the MLNs at 14 post-infection. Peripheral IFN-γ in the serum was detected at 7 days post-infection (Dawson et al., 2005). The phenotype of PBMCs was characterized after oral infection with *T. gondii* oocysts. A temporal decrease in the percentage of CD4^+^ cells on day 6 post-infection and a significant increase in the percentage of CD8^+^ cells was observed in the second week of infection. An early activation of cells was also detected in the first week of infection by an increase in expression of activation markers CD25 and SLA-DQ (Solano Aguilar et al., 2001). At 6 weeks post-oral infection with *T. gondii* oocysts, transcription analysis in MLNs, retropharyngeal lymph node and spleen, showed significant increase of IFN-γ transcripts both in spleen and retropharyngeal lymph node and of the T-cell surface marker CD8α, the natural killer (NK) cell marker CXCR3 (C-X-C Motif Chemokine Receptor 3), and the adaptor protein MyD88 in the retropharyngeal lymph node (Bartley et al., 2019). Recently, transcriptome sequencing analysis of different pig tissues (liver, spleen, cerebral cortex, lung, and MLNs) was used to characterize the porcine tissue transcriptional landscapes 6 and 18 days post-oral infection with *T. gondii* oocysts (He et al., 2019). *T. gondii* infection causes differential expression of transcription factors, such as Irf1, Irf8, Stat1, and Stat3. Cytokines (38) and cytokine receptor-related transcripts (21) that were differentially expressed were also identified. Upregulation of these genes can increase chemotaxis of immune cells, including dendritic cells (DCs), NK cells, macrophages, and T cells that can be regulated by C-X-C Motif Chemokine Ligand (CXCL)9, CXCL10, and CXCR3 signaling pathways contributing to the pig immune response to *T. gondii* infection. Genomic hotspots encoding 24 host genes significantly correlated with *T. gondii* load were also identified some of them have known anti-*T. gondii* activity, such as Gbp1, Gbp2, Gbp7, Batf2, and Tap1.

Following oral infection of pig with *T. gondii* cysts, IFN-γ secretion by mediastinal and duodenal lymph node cells stimulated with *T. gondii* antigens was observed from day 8 post-infection onwards while jejunal and ileal lymph node cells did not secrete IFN-γ (Rahman et al., 2020). Restimulated PBMC cells secreted IFN-γ from day 14 post-infection onwards (Rahman et al., 2020). Similarly, IFN-γ mRNA expression was observed from week 2 to 5 post-oral infection with *T. gondii* cysts (Verhelst et al., 2011). During the same follow-up period, IL-10 and IL-4 responses were not increased (Verhelst et al., 2011; Verhelst et al., 2015). In a study from 1 month to 4 months post-oral infection with *T. gondii* cysts, PBMC cells restimulated with *T. gondii* antigens produced IFN-γ mRNA from 1 month to the end of the experiment, while IL-10, IL-12 and IL-17 mRNA productions were not detected (Jennes et al., 2017). The IFN-γ production by restimulated PBMCs was correlated with the infection dose and predominantly brought about by CD3^+^ CD4^-^ CD8^+^ T lymphocytes (Jennes et al., 2017).

*T. gondii* oral infection with oocyst or cysts promotes mucosal and systemic humoral responses. The presence of anti-*T gondii* IgG antibodies in sera of pig infected orally with *T. gondii* oocysts or cysts was detected within 2 weeks post infection (Verhelst et al., 2011; Burrells et al., 2015; Basso et al., 2017; Campero et al., 2020; Rahman et al., 2020). Anti-*T. gondii* IgM antibodies were detected 2 weeks post-infection and disappeared by week 6 post-infection with *T. gondii* cysts (Verhelst et al., 2011). A specific IgG antibody response was found sixty days after oral infection with *T. gondii* oocysts in pig aqueous humor (Garcia et al., 2008). Anti-*T. gondii* IgA and IgG antibodies were also detected in oral fluid, a combination of saliva and serum transudates from capillaries in the oral mucosa and gingival tissues, 1.5 to 4 weeks post-infection with *T. gondii* oocysts (Campero et al., 2020).

### Human Immunity

Analysis of immune responses in human is largely confined to the peripheral blood and, there is no way to examine immune responses at the site of initial infection. Identification of susceptibility alleles and human primary immune-deficiencies that are associated with *Toxoplasma* infection provide also information about what is needed for protection (Ngô et al., 2017; McLeod et al., 2020).

The innate immune system is the first to respond to infection with production of chemokines, interleukins and growth factors. Monocytes infected with *T. gondii* tachyzoites released Alarmin S100A11, leading to the RAGE dependent induction of the chemokine C-C Motif Ligand 2 (CCL2) a major chemokine required for monocyte-mediated immunity (Safronova et al., 2019). Additionally, in infected monocytes (isolated from healthy donors) the inflammasome sensor NLRP3 (Gov et al., 2017) is induced leading to IL-1β production. Moreover, the inflammasome sensor NLRP1 has also been implicated (Witola et al., 2011). Susceptibility to human toxoplasmosis was found to map to alleles of the NALP (NLRP1) gene and NALP1 was shown to contribute to the control of parasite growth in a human monocyte cell line (Witola et al., 2011). Monocytes and dendritic cells (CD1c^+^) produced IL-12 and TNF-α in response to phagocytic uptake of live parasites (Tosh et al., 2016) whereas neutrophils produced both IL-12 and TNF-α in response to *T. gondii* antigen stimulation (Bliss et al., 1999).

Up-regulation of CD80, CD86, and CD40 on dendritic cells infected with *T. gondii*, have important implication for the initiation of a T cell response because T cell production of INF-γ in response to *T. gondii-*infected DC is dependent on CD40-CD40L and CD80/CD86-CD28 signaling (Subauste and Wessendarp, 2000). Recently, a morphological and immunohistochemical evaluation of inflammatory cells in axillary lymph nodes from a symptomatic acute toxoplasmosis which self-resolved, demonstrated the presence of M1 macrophages and of T helper 1 lymphocytes (De-Luca et al., 2018).

In chronic asymptomatic individuals, the PBMCs’ immune responses against *T. gondii* antigens are predominantly characterized by high levels of IFN-γ (Prigione et al., 2006; Meira et al., 2015) and most *T. gondii*-specific CD4^+^ T-cell clones obtained from PBMCs produced IFN-γ (Saavedra and Hérion, 1991; Daubener et al., 1995). Polymorphisms in the human endoplasmic reticulum-associated aminopeptidase (ERAAP) gene ERAP1 were associated with susceptibility to human congenital toxoplasmosis, emphasis the importance of MHC Class I antigen processing in humans in response to *T. gondii* (Tan et al., 2010).

In addition to activating T cell-mediated immunity, IFN-γ activates different mechanisms to control intracellular *T. gondii* that seems to depend on specific host cell type. They include increased production of ROs (Murray et al., 1985), tryptophan limitation due to the upregulation of IDO (Pfefferkorn, 1984), the sequestration of iron (Dimier and Bout, 1998), K63-linked ubiquitination for endosomal destruction (Clough et al., 2016), noncanonical autophagy (Selleck et al., 2015). Recently, in IFN-γ-activated infected human macrophages, GBP1 recruitment to *T. gondii* PVs was shown to promote the lysis of *T. gondii* vacuoles and parasite membranes, releasing *T. gondii* DNA to trigger activation of the AIM2 inflammasome (Fisch et al., 2019).

Oral infection with *T. gondii* cysts or oocysts stimulates a humoral immunity used for the diagnosis of *Toxoplasma* infection. The humoral response involves IgG, IgM, IgA and IgE specific antibodies which may be detected in serum, tears, saliva, cerebrospinal fluid, colostrum and milk. Serum IgA and IgM antibodies specific to *T. gondii* are found in the serum during the first week following infection. Specific IgG antibodies may be detected 1 to 3 weeks after the initial rise in IgM levels and persists lifelong at residual titers (Correa et al., 2007; Robert-Gangneux and Dardé, 2012).

### Mouse Immunity

The immune response to *T. gondii* has been extensively studied in mouse models. The mucosal immune system of the intestine is the front line of defense against *T. gondii.* Mucosal immunity involves the complex coordination of cells and cytokines to enable control *T. gondii* infection without permitting emergence of proinflammatory immunopathology (Cohen and Denkers, 2015). Following oral infection, infected enterocytes secrete chemokines recruiting immune cell subsets. Neutrophils are one of the first cell types to arrive at the site of infection; they participate in the recruitment and activation of other immune cells such as macrophages and dendritic cells.

The innate event that initiates the production of IFN-I and proinflammatory cytokines including TNF-α and IL-12, is the recognition of *T. gondii via* pattern recognition receptors (PRRs). Among them, Toll-like receptors (TLRs) play a central role. Mice lacking MyD88, an adaptator molecule that acts downstream of TLR and IL-1 receptor family members are severely impaired in IL-12 production and failed to control the parasite after oral or systemic infection (Scanga et al., 2002; Sukhumavasi et al., 2008). Significant reduction of IFN-β was also observed in MLNs 5 days following oral infection with *T. gondii* cysts of mice lacking MyD88 (Han et al., 2014). *T. gondii* can stimulate innate immunity *via* multiple TLRs. *T. gondii* profilin is recognized by TLR 11/12, resulting in Myd88-dependent IL-12 production, a dominant mechanism driving IL-12 production in mice following intraperitoneal infection (Yarovinsky et al., 2005; Koblansky et al., 2013) or oral infection (Benson et al., 2009). TLR9, 2 and 4 have also been shown to be involved in the development of efficient IFN-γ responses by T cells in the lamina propria after oral infection with *T. gondii* cysts (Minns et al., 2006; Benson et al., 2009) and commensal bacteria are responsible for the activation of these TLRs (Benson et al., 2009). Another study, using deficient TLR4 or TLR2 deficient mice also showed that TLR4 deficient mice but not TLR2 deficient mice, had a higher number of brain cysts 35 days post oral infection with *T. gondii* cysts with a decrease of proinflammatory response 5 days after infection than wild type control mice. In contrast, following intraperitoneal infection TLR4 deficient mice had almost identical number of brain cysts and produced similar proinflammatory cytokines compared to wild type control mice (Furuta et al., 2006). As other *T. gondii* candidates able to activate TLRs, tachyzoite-derived GPIs have been shown to induce TLR2 and TLR4 dependent production of TNF-α in murine macrophages (Debierre-Grockiego et al., 2003) and recently microneme proteins 1 and 4 were also reported to stimulate IL-12 secretion in a TLR2/4-dependent manner in bone-marrow-derived DCs (Sardinha-Silva et al., 2019). In addition to the TLRs, *T. gondii* is also detected by C-C motif chemokine receptor 5 (CCR5) expressed on DC cells. *T. gondii* cyclofilin 18 is recognized by CCR5, stimulating IL-12 expression through activation of the G protein α I 1 family and MAPK (Aliberti et al., 2000). Furthermore, *T*. *gondii* triggers inflammasome activation *via* the inflammasome sensors NLRP1 and 3 in a caspase-1-dependent manner resulting in IL-1β and IL-18 production (Ewald et al., 2014; Gorfu et al., 2014; López-Yglesias et al., 2019). A recent study, demonstrated that *in vivo* inflammasome activation in response to *T. gondii* occurs in CD8α^+^ DCs, inflammatory monocytes and neutrophils and suggested that a third sensor for *in vivo* *T. gondii* detection must exist in addition to NLRP1 and NLRP3 (Kupz et al., 2020).

The cytokine IL-12 controls the infection through initiation of IFN-γ production and is a key cytokine that links innate and adaptive immune system (Gazzinelli et al., 1994; Yap et al., 2000). Using bicistronic IL-12- yellow fluorescent protein reporter mice, it was shown that CD11c^+^MHCII^+^F4/80^-^ dendritic cells, F4/80^+^ macrophages, and Ly6G^+^ neutrophils were the dominant cellular sources of IL-12 in the peritoneal cavity of mice infected intraperitoneally with *T. gondii* tachyzoites (Mercer et al., 2020). Dendritic cells contributed to the largest IL-12 positive population (Mercer et al., 2020). Furthermore, CD8α^+^ DCs were identified as the key source of IL-12 as shown by the acute susceptibility and decreased IL-12 and IFN−γ production of *Batf3* ^−/−^ mice, which selectively lack only lymphoid resident CD8α^+^ DCs and related peripheral CD103^+^ DCs, during early systemic infection with *T. gondii* tachyzoites. This susceptibility was reversed by administration of IL-12. Oral infection of Batf3^−/−^ mice demonstrated a similar susceptibility of these animals to *T. gondii* infection, with acute lethality and a failure to control parasite replication. Specifically, gut CD103^+^ DCs are also absent in the Batf3^−/−^ mice and may play a role during oral infection (Mashayekhi et al., 2011). A multiparameter flow cytometry identified in lymphoid tissues from intraperitoneally *T. gondii* infected mice an expansion and activation of both cDC subsets and plasmacytoid DCs and expansion of plasmacytoid DCs was also observed following oral infection (Pepper et al., 2008). Both plasmacytoid DCs maturation and IL-12 cytokine production are dependent on TLR11 (Pepper et al., 2008).

As a proinflammatory cytokine, IL-12 stimulates IFN-γ production, a key cytokine for control of acute and chronic infection (Suzuki et al., 1989; Suzuki and Remington, 1990; Gazzinelli et al., 1994). IL-12 stimulates intestinal innate lymphoid cells (ILC)-1 to produce IFN-γ and TNF-α in response to *T. gondii* oral infection. ILC1s are the main producers of IFN-γ and TNF-α compared to cNK cells and NKp46^+^ NK1.1^+^ ILC3s and mice lacking ILC1 (Tbx21^-/-^) failed to control parasite replication in the small intestine and to recruit inflammatory monocytes (Klose et al., 2014). IL-12 triggers the production of IFN-γ later by T lymphocytes (Gazzinelli et al., 1994; Shirahata et al., 1994; Yap et al., 2000). CD4^+^ T lymphocytes, recruited in dependence upon their expression of the chemokine receptor CXCR3 in the intestine, mediate activation of intestinal mucosa inflammatory monocytes *via* secretion of IFN-γ (Cohen et al., 2013).

An IL-12 independent production of IFN-γ by neutrophils that is regulated by TNF and IL-1β has been reported and selective elimination of neutrophils in TLR11 deficient mice infected intraperitoneally with *T. gondii* cysts resulted in acute susceptibility (Sturge et al., 2013). Inflammatory monocytes recruited by chemokine CCL3 produced in the intestinal mucosa by ICLs cells in dependence upon IL-18 in the intestine are associated with the ability to suppress early parasite replication at the site of infection (Dunay and Sibley, 2010). Furthermore, inflammatory monocytes have been identified as the major source of IFN-β in MLNs after oral infection with *T. gondii* cysts and mice lacking the receptor for type I IFN‐1 showed higher parasite loads and reduced survival (Han et al., 2014). Expression of IFN-β by inflammatory monocytes requires phagocytic uptake of parasites as well as signaling through TLR4 and MyD88 (Han et al., 2014).

A variety of cells respond to IFN-γ stimulation. IFN-γ signaling proceeds through activation of the signal transducer and activator of transcription 1 (STAT1). STAT1 upregulates the production of effector molecules such as NO and ROS in hematopoietic cells that lead to inhibition of intracellular parasite growth (Hunter and Sibley, 2013). IFN-γ also triggers the induction of immunity-related GTPase (IRG) proteins and GBPs, both in non-myeloid and myeloid cells to damage the PV membrane (PVM) (Howard et al., 2011; Kravets et al., 2016). IRG and GBP families are important factors in cell-autonomous immunity in mice. IFN-γ plays key roles not only in activating effector cells to kill the parasite but also in stimulating a production of mediators to recruit immune T cells from the periphery to the brain where tachyzoites transform into cysts to establish chronic infection (reviewed in Suzuki, 2020).

Adaptative immunity relies on the migration of antigen presenting cells (APCs) from the site of infection to secondary lymphoid organs and the activation of resident APCs through exposition to *T. gondii* antigens and on their ability to present antigens and to activate B cells, CD4^+^ and CD8^+^ T cells. Protection against *T. gondii* infection is mainly attributed to cell-mediated immunity and both CD4^+^ and CD8^+^ T cell subtypes are involved in the protection (Suzuki and Remington, 1988; Gazzinelli et al., 1991; Gazzinelli et al., 1992; Casciotti et al., 2002). Specific T lymphocytes act either as cytokine producer cells that help infected cells to kill the parasite or as cytotoxic cells that destroy infected cells. If IFN-γ production by infiltrated CD4^+^ and CD8^+^ T cells in the brain is required for the prevention of cerebral tachyzoite growth, anti-cyst activity of CD8+ T cells requires perforin in conjunction with microglia and macrophages help. CXCR3, CXCR6, and IL-18R are involved in recruitment and activation of microglia and macrophages to the T cell-attacked cysts for their elimination (Lutshumba et al., 2020). Peptide repertoire presented by MHC class I molecules to CD8^+^ T cells is shaped by ERAAP. ERAAP-deficient H-2d mice generated significantly lower frequencies of immunodominant HF10-specific CD8^+^ T cells and were consequently not protected from *T. gondii* infection (Blanchard et al., 2008).

Oral infection with *T. gondii* cysts also stimulates humoral immunity, in the digestive tract, the serum and milk (Chardes et al., 1990). The role of antibodies on resistance against *T. gondii* remained unclear until the use of B-cell-deficient mice (Kang et al., 2000; Johnson and Sayles, 2002). The results indicated the importance of the B-cell antibody response in preventing persistent proliferation of tachyzoites in the brain and lung during the chronic phase of infection.

### *T. gondii* Antigens Recognized by Host Adaptive Immunity

#### Humoral Response

Infection is initiated by ingestion of oocysts or tissue cysts containing sporozoites and bradyzoites respectively. Around 2 days after ingestion and invasion of intestinal epithelial cells both convert into dividing tachyzoites which disseminate (acute infection), then tachyzoites convert to bradyzoites which persist the host’s lifetime (chronic infection). Tachyzoites, sporozoites and bradyzoites have unique as well as shared antigens (Fritz et al., 2012; Nadipuram et al., 2020). Some antigens are no longer expressed by the parasite, in particular oocyst/sporozoite specific proteins in intermediate hosts. Specific bradyzoite proteins may be detected at the mucosal level at the beginning of infection and at the systemic level, latter during the induced chronic toxoplasmosis.

Serum IgG antibodies specific to *T. gondii* are mainly directed against tachyzoite/bradyzoite antigens. Most of the *T. gondii* antigens carrying B-cell epitopes have been identified after the screening of *T. gondii* cDNA libraries or high throughput protein microarray screening approaches (Beghetto et al., 2001; Hill et al., 2011; Liang et al., 2011; Döşkaya et al., 2014; Felgner et al., 2015; Döşkaya et al., 2018; Arranz-Solís et al., 2019) with sera from infected animals or infected humans. These antigens included rhoptry (ROP1, 2, 5, 6, 8, 18), dense granules (GRA1, 2, 3, 4, 5, 6, 7, 8, 9, 14), micronemes (MIC1, 2, 3, 4, 12), M2AP, AMA1, ROM5, MAG, P68, (Felgner et al., 2015; Döşkaya et al., 2018; Ybañez et al., 2020), surface proteins (SAG1, SAG2, SAG2A (SRS34A), SAG2C (SRS49D), SRS52A, SRS29A, SRS13). Serum IgG antibodies specific to bradyzoite BAG1 and MAG1 proteins, were detected early after infection in human sera (Di Cristina et al., 2004) and in mice orally infected with cysts (Hester et al., 2012). Serum IgG antibodies specific to oocyst/sporozoite proteins, namely TgOWP8, TgERP and TgCC5A were detected in sera from human and animals infected with oocysts (Hill et al., 2011; Santana et al., 2015; Liu et al., 2019). These proteins were proposed as new tools to identify the parasite stage of infection, allowing better understanding of sources of *T. gondii* infection in animals and humans.

The presence of mucosal antibodies measured in saliva, intestinal fluids, milk and tears has been reported (Correa et al., 2007). Salivary IgA antibodies specific to sporozoite TgERP protein, were detected in human infected with oocysts (Mangiavacchi et al., 2016). Oral infection with *T. gondii* oocysts induced production of anti-SAG1 IgG and IgA specific to tachyzoite stage in human saliva (Chahed Bel-Ochi et al., 2013) and oral fluids from pigs (Campero et al., 2020). In cat, oral infection with oocysts induced specific antibodies in sera, intestinal secretions, and fecal extracts not only to *T. gondii* tachyzoites, but also to bradyzoites, sporozoites, and enteroepithelial stages (Rush et al., 2001). The IgA from intestinal secretions bound to antigens on all enteroepithelial stages and to a less degree to bradyzoites and sporozoites but did not bind to tachyzoites whereas serum IgG bound to tachyzoites, bradyzoites, sporozoites, and enteroepithelial stages of *T. gondii* and serum IgA bound strongly to enteroepithelial stages but only weakly to tachyzoites and bradyzoites, suggesting a compartmentalization of the humoral response.

#### Cellular Response

Several endogenous CD8^+^ *T. gondii*–derived epitopes have been identified to date (**Table 2**). Search for candidate CD8^+^ T cell epitopes restricted by specific HLA molecules was based on several bioinformatic algorithms, and then binding affinity assays. The selected peptides were tested for their ability to elicit IFN-γ production by human PBMCs from seropositive (but not seronegative) people using an IFN-γ ELISPOT assay. For this purpose, amino acid sequences from *T. gondii* tachyzoite, bradyzoite and sporozoite secreted or surface antigens were selected. These sequences included those of SAG1, SAG2C, SAG2D, SAG2X, SRS52A, SAG3, MIC1, MICA2P, GRA5, GRA6, and GRA3 for HLA-A02 specific peptide-binding, SAG1, SAG2C, SRS52A, GRA5, GRA6, and GRA7, for HLA-A03 specific peptide-binding and GRA3 and GRA7 for HLA-B07 specific peptide-binding (Tan et al., 2010; Cong et al., 2010; Cong et al., 2011; Cong et al., 2012). Two peptides for HLA-A02 specific peptide binding were also reported by Cardona et al. (2015), one from a putative ER-retrieval receptor (Ter1p) and another one from a Glutamicoxaloacetic transaminase (Got1). Both proteins have peptide signal and transmembrane domains and predicted to be cleaved at the proteasome. They are involved in parasite metabolic events. Furthermore a CD8^+^ T cell clone derived from a person with chronic toxoplasmosis was shown to kill EBV-Ya cells infected with RH tachyzoites or pulsed with SAG1^289-297^ (SPEKHHCTV) but not uninfected and non-pulsed EBV-Ya cells. The cytotoxicity was blocked with anti-HLA-A02 and anti-CD8 monoclonal antibodies (Yano et al., 1997).

**Table 2:** HLA-restricted *T. gondii* CD8^+^ T cell epitopes.

Gene ID^1^	Protein^2^	Peptide sequences	Immunogenicity^3^in mice (IFN-γ)	References
**HLA-A2**	**Average population**	**coverage: 25%**		
227280 286450	GRA3 (T, B) GRA5 (T, B, SZ)	GRA3 ^25–33^ (FLVPFVVFL) GRA5 ^82-90^ (GLAAAVVAV)	+ +	Cong et al., 2011 Cong et al., 2011
275440	GRA6 (T, B, SZ)	GRA6 ^24–32^ (VVFVVFMGV) GRA6 ^29–37^ (FMGVLVNSL)	− +	Cong et al., 2011 Cong et al., 2011
233460 207160 207150	SAG1/SRS29B (T, SZ) SAG2C/SRS49D (B) SAG2D/SRS49C (B)	SAG1^289-297^ (SPEKHHCTV SAG2C ^38–46^ (FLSLSLLVI) SAG2D ^180–189^ (FMIAFISCFA)	ND + +	Yano et al., 1997 Cong et al., 2011 Cong et al., 2011
207140	SAG2X/SRS49B (T)	SAG2X ^44–52^ (FVIFACNFV) SAG2X ^351–359^ (FMIVSISLV)	+ +	Cong et al., 2011 Cong et al., 2011
308020	SAG3/SRS57 (T, B, SZ)	SAG3 ^375–383^ (FLLGLLVHV) SAG3 ^136–144^ (FLTDYIPGA)	+ +	Cong et al., 2011 Cong et al., 2011
315320 291890 214940 295460 206350	SRS52A MIC1 (T, SZ) MIC2-M2AP (T, B, SZ) Got1 family protein Ter1p	SRS52A ^12–20^ (ITMGSLFFV) MIC1 ^9–17^ (VLLPVLFGV) MICA2P ^11–19^ (FAAAFFPAV) Got1 ^26-34^ (FLDRALLTL) Terp1 ^184-192^ (FLADLLHSV)	+ + − ND ND	Cong et al., 2011 Cong et al., 2011 Cong et al., 2011 Cardona et al., 2015 Cardona et al., 2015
**HLA-A3**	**Average population**	**coverage: 24%**		
286450 275440 203310 233460 207160 315320	GRA5 (T, B, SZ) GRA6 (T, B, SZ) GRA7 (T, B, SZ) SAG1/SRS29B (T, SZ) SAG2C/SRS49D (B) SRS52A	GRA5^89-98^ (AVVSLLRLLK) GRA6^164-172^ (AMLTAFFLR) GRA7^134-142^ (RSFKDLLKK) SAG1^224-232^ (KSFKDILPK) SAG2C^13-21^ (STFWPCLLR) SRS52A ^250-258^ (SSAYVFSVK)	+ + − + + +	Cong et al., 2010 Cong et al., 2010 Cong et al., 2010 Cong et al., 2010 Cong et al., 2010 Cong et al., 2010
**HLA-B7**	**Average population**	**coverage: 30%**		
227280 203310	GRA3 (T, B) GRA7 (T, B, SZ)	GRA3 ^27–35^ (VPFVVFLVA) GRA7 ^20–28^ (LPQFATAAT)	− +	Cong et al., 2012 Cong et al., 2012

^1^TGME49_Gene identifier according to ToxoDB.org. (ME49 type II strain).

^2^Identity of protein assigned by ToxoDB.org, stage specificity: tachyzoite (T), bradyzoite (B), sporozoite (SZ).

^3^Splenic T cells were isolated from HLA-A2, A3 or B7 supertype mice after peptide immunization and tested for their ability to generate IFN-γ response to peptide.

ND, not done.

+, IFN-g production; -, no production of IFN-g.

In the search of parasite antigens involved in CD4^+^ T cells response, T cell clones derived *in vitro* from chronically infected healthy persons or PBMCs were stimulated with a range of selected recombinant *T. gondii* proteins (or fragment of proteins). Proliferative responses and/or cytokine production were obtained for SAG1 and GRA2 in a HLA-DR-restricted manner (Prigione et al., 2000), for MIC2, MIC3, MIC4 and M2AP (Beghetto et al., 2005), for SAG1, GRA1, GRA6, and GRA7 (Fatoohi et al., 2002), SAG1, ROP1, GRA8 and MAG1 (Silva-Gutierrez et al., 2018) and for BAG1 and MAG1 (Di Cristina et al., 2004). In one study, the epitope peptide DPw4 restricted (ROP2^197-216^: TDPGDVVIEELFNRIPETSV) recognized by a T cell clone specific for ROP2 was further defined using several algorithms followed by *in vitro* stimulation of the T cell clone (Saavedra et al., 1996).

To our knowledge, such approaches to identify T cell epitopes have not been undertaken for sheep, pigs and cats. Plenty of *T. gondii* antigens are recognized by specific antibodies, in all host species. As B cell activation and initiation of the humoral immune response to protein antigens requires both B and T cell involvement, this suggests that these targeted antigens potentially contain both CD4^+^ T cell T and B epitopes.

## Immunity Against *T. gondii*: The Parasite Perspective

The mechanisms of *T. gondii* persistence are studied mostly at the tachyzoite stage. *T. gondii* has developed a multitude of strategies partially strain-dependent, host cell-type specific and host-species dependent (reviewed in Hakimi et al., 2017; Lima and Lodoen, 2019; Poncet et al., 2019; Zhu et al., 2019; Wong et al., 2020).

*T. gondii* manipulates host immunity through secretion of proteins, mainly ROP and GRA proteins that modify host transcriptional programs or signaling pathways. Through ROP18/ROP5/ROP17/GRA7 complex, type I strains of *T. gondii* inactivate host IRGs to evade the IFNγ-inducible GTPase-mediated host defense which plays a crucial role in the destruction of the PVM in a murine model. ROP54 in type II strain was also shown to interfere with the noncanonical IFN-γ dependent autophagy pathway by restricting immune loading of GBP2 to evade the GBP2-mediated immune response (Kim et al., 2016). Type I ROP18 has been shown to phosphorylate the transcription factor ATF6β leading to downregulation of CD8 T cell activation (Yamamoto and Takeda, 2012) and to inhibit the host NF-κB pathway by promoting p65 degradation (Du et al., 2014). The dense granule-resident effector, called TEEGR (Toxoplasma E2F4-associated EZH2-inducing Gene Regulator) has been shown to suppress NF-κB regulated TNF-α cytokine signaling in a type II strain (Braun et al., 2019). GRA18 (conserved across the *Toxoplasma* lineages) induces β-catenin-dependent genes associated with anti-inflammatory responses, including CCL17 and CCL22 chemokines in murine infected macrophages (He et al., 2018). *Toxoplasma* inhibitor of STAT1-dependent transcription (TgIST) (type I, II), which represses STAT1-dependent promoters (Gay et al., 2016; Olias et al., 2016) could be the effector responsible for the dysregulation of MHC II molecule expression in infected host cells (Poncet et al., 2019). ROP16 (type I, III) phosphorylates STAT3 and STA6, mainly leading to a decrease in IL-12 and Th1 response (Saeij et al., 2007; Yamamoto et al., 2009; Ong et al., 2010).

Contribution of these specific modulatory effectors in the chronic infection remains poorly investigated. Recently, Mayoral et al. (2020) showed that despite a decline in nuclear accumulation, TgIST still mediated suppression of IFN-γ signaling after prolonged infection with bradyzoites, and provided evidence that TgIST is exported beyond the early stages of host cell infection. TgPL1, a patatin-like phospholipase 1, localized within vesicles inside the tachyzoite, is localized to the PV and cyst wall in the encysted bradyzoite stage. TgPL1 was previously determined to be necessary for tachyzoites to suppress NO synthesis and prevent degradation in activated macrophages (Mordue et al., 2007; Tobin and Knoll, 2012). Contrary to mice infected with wild type parasites, mice infected with parasites lacking TgPL1 maintain high cytokine levels and do not display toxoplasmic encephalitis symptoms, suggesting that TgPL1 plays a role in the maintenance of chronic *T. gondii* infection (Tobin Magle et al., 2014).

Two specific modulatory effectors identified in type II strains namely GRA15 and GRA24 have an opposite effect, by promoting of a proinflammatory signal. GRA24 in type II strain activates p38 MAPK signaling pathway by prolong autophosphorylation and nuclear translocation of host cell p38a, leading to production of proinflammatory cytokine IL-12 in both infected macrophages and dendritic cells (Kim et al., 2005; Braun et al., 2013; Ten Hoeve et al., 2019). IL-12 produced by DCs plays a critical role in controlling acute *Toxoplasma* infection in mice (Mashayekhi et al., 2011), but at the same time, infected dendritic cell migration is maintained to contribute to efficient dissemination of the parasite (Ten Hoeve et al., 2019). GRA24 is not secreted beyond the PVM, is not localized to the host cell nucleus and instead accumulates within the *in vitro* tissue cysts (Krishnamurthy and Saeij, 2018). These observations suggest that GRA24 contributes to effective control of acute *T. gondii* infection and promotes the conversion of parasite into bradyzoites and persistence at the chronic infection stage. GRA15 type II activates the NF-κB pathway through interaction with tumor necrosis factor receptor associated factors (TRAFs), which are adaptor proteins functioning upstream of the NF-κB transcription factor (Sangaré et al., 2019), leading to human monocyte production of IL-1β (Gov et al., 2017), induction of IL-12 secretion by murine macrophages (Rosowski et al., 2011), and upregulation of the CD40 cell surface receptor on murine macrophages, which leads to IL-12 production (Morgado et al., 2014). The role of GRA15 during the chronic phase of infection has not been investigated.

The growing list of parasite effectors responsible for the activation, inhibition, and interaction of signaling pathways involved in the persistence of *T. gondii* in host cells will permit the identification of the strategies developed by *T. gondii* to limit its virulence thereby promoting the survival of its host and the formation of tissue cysts.

## Toward Effective Vaccines Against *T. GONDII*

### Livestock Animals and Cats

#### Live Vaccines in Livestock Animals and Cats

The recent development of new techniques based on genetic engineering allowed the explosion of strategies for vaccine development. Over the last decades, the field of vaccinology has been exploring safer vaccines by creation of recombinant immunogenic proteins, naked DNA and viral/bacterial recombinant vectors. Although promising, the strategies based on single or few antigens are less efficacious than attenuated live *T. gondii* strains. Recombinant *T. gondii* strains present all the diversity and complexity of *T. gondii* antigens, and enable appropriate processing and presentation of *T. gondii* antigens in the context of MHC class I antigens, CD8^+^ T cells and IFN-γ being key elements of a protective immune response. Until now, most of the live attenuated *T. gondii* vaccines are tachyzoites, the only stage easy to produce *in vitro* and in large quantities.

The first one, the S48 strain live vaccine, resulted in one commercially available vaccine (Toxovax), for protection against congenital toxoplasmosis in sheep. The S48 strain has lost the ability to differentiate into bradyzoite and tissue cysts (Buxton, 1993) as a result of being passaged for many years in laboratory mice. In ewes, subcutaneous inoculation of 10^5^ tachyzoites of the S48 strain, induced both cellular (CD4^+^ and CD8^+^ T cells) and humoral responses (Innes and Wastling, 1995; Wastling et al., 1995) and conferred significant protection against abortion after oral challenge with *T. gondii* oocysts (Buxton et al., 1993). Studies have been carried out in lambs and pigs inoculated subcutaneously with the S48 strain, to evaluate its ability to reduce parasite burden in meat and thus improving food safety. It was found that the S48 strain protected against the development of tissue cysts following an oral challenge with *T. gondii* cysts (Katzer et al., 2014; Burrells et al., 2015). A chemically-induced mutant *T. gondii* strain that lacks the capacity to form oocysts in cat but undergoes both schizogony and gametogony in the intestine of cats after ingestion of tissue cysts (Dubey, 2017), the T-263 strain, was found to be effective in inducing immunity in cats (Frenkel et al., 1991; Freyre et al., 1993). Kittens orally inoculated with tissue cysts of the T-263 strain did not shed oocyst when challenged orally with tissue cysts of a heterologous normal strain (84% of the kittens) (Frenkel et al., 1991). The abilities of *T. gondii* T-263 tissue cysts, bradyzoites released from tissue cysts, and tachyzoites, to confer protection, were compared (Freyre et al., 1993). Orally administered bradyzoites of T-263 strain, either contained in intact tissue cysts or liberated from cysts, induced immunity to oocyst shedding. In contrast, tachyzoites did not completely protect against oocyst shedding, even when delivered directly to the duodenum (Freyre et al., 1993). A 3 years field trial on swine farms was conducted to determine the effectiveness of cat vaccination with bradyzoites of the T-263 strain, in reducing the prevalence of *T. gondii* in finishing pigs (Mateus-Pinilla et al., 1999). Under field conditions, cat vaccination was able to significantly reduce *T. gondii* infection in finishing pigs, suggesting that cat vaccination could contribute to improve food safety.

**Figure 1:**
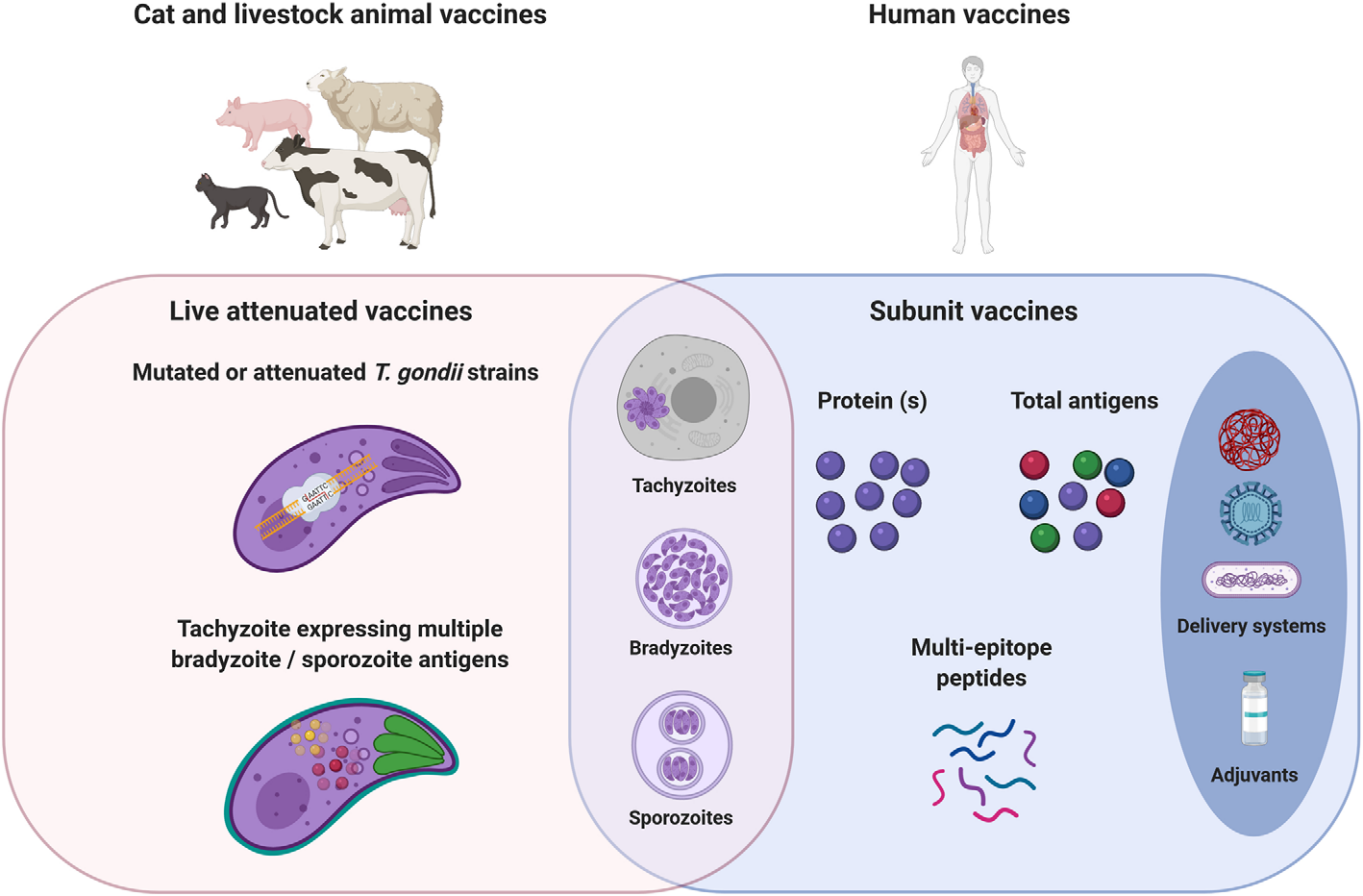
Cat, livestock, and human toxoplasmosis vaccine strategies. *Toxoplasma* infection is acquired by three stage forms: tachyzoite, bradyzoite (cyst), or sporozoite (oocyst). In a one health vaccination strategy, the objectives would be the prevention of congenital disease in both women and livestock, the prevention/reduction of *T. gondii* tissue cysts in food-producing animals; and oocyst shedding in cats. Live mutated or attenuated *T. gondii* tachyzoite stage vaccines are mostly used for cat and livestock animals. The development of tachyzoites expressing multiple bradyzoite/sporozoite antigens could therefore improve the immunogenicity of live vaccines, whatever the *T. gondii* stage. Live vaccines would require passing considerable regulatory hurdles that may impede their use in human. Safer subunit vaccines have been explored by the generation of recombinant immunogenic proteins. The use of *T. gondii* extract allows vaccination with all the diversity and complexity of *T. gondii* antigens. Subunit vaccines need delivery systems and adjuvants to improve antigens bioavailability and stimulate protective cellular immune responses to *T. gondii.* Studies aiming at the identification of CD8^+^, CD4^+^, and B cell specific *T. gondii* epitopes are still needed to rationally design an efficient multi-epitope vaccine engineered to focus both T and B cells responses toward a set of critical epitopes selected from a wide range of antigens in order to diversify the scope of the induced immune responses.

In recent years, the availability of the complete *T. gondii* genome sequence and genetic engineering methods allowed to develop genetically attenuated *T. gondii* strains, mostly by gene deletions. These new attenuated strains would be safer than S48 and T263 strains obtained after passage for many years in laboratory mice and chemical-induced mutations respectively. The first to be tested in sheep and cat was the strain called Mic1-3KO obtained by deletion of *MIC1* and *MIC3* genes in the *T. gondii* RH strain (Cérède et al., 2005). These genes encode microneme proteins involved in adhesion of the parasite to host cells (Fourmaux et al., 1996; Garcia-Réguet et al., 2000). The simultaneous disruption of the two genes was shown to impair invasion of cells *in vitro* and to impair virulence in mice (Cérède et al., 2005). The Mic1-3KO strain confers protection against chronic and congenital toxoplasmosis in outbred mice (Ismael et al., 2006). Ewes inoculated either subcutaneously or intraperitoneally with tachyzoites of the Mic1-3KO strain developed protection against *T. gondii* abortion following oral challenge with *T. gondii* oocysts (Mévélec et al., 2010). Mic1-3KO was as effective as the S48 strain. Moreover, preliminary results showed the potential of Mic1-3KO to reduce the development of tissue cysts in lambs born to vaccinated ewes. However, when administrated to cat subcutaneously or orally in a gastro-resistant solution to resist intestinal passage, Mic1-3KO did not prevent cats from oocysts shedding following oral challenge with *T. gondii* oocysts (Le Roux et al., 2020). Recently, a HAP2-deficient *T. gondii* strain was created using a CRISPR/Cas9 strategy in the CZ clone H3 *T. gondii* (type II strain). This HAP2-deficient *T. gondii* strain generates oocysts that fail to produce sporozoites (oocysts do not undergo meiosis). Oral administration of HAP2-deficient *T. gondii* tissue cysts to cats was shown to totally prevent oocyst excretion after oral infection with tissue cysts from wild-type *T. gondii*, demonstrating that this mutant could be a transmission-blocking vaccine (Ramakrishnan et al., 2019) (**Figure 1**).

#### Vaccination Perspectives for Cats and Livestock Animals

Oral vaccination of cats with bradyzoites of T263 strain led to effective prevention of oocyst excretion (Freyre et al., 1993). Bradyzoites that invade the feline intestinal epithelium differentiate into five morphologically distinct types of schizonts (Dubey and Frenkel, 1972). It is known that the enteric parasite cycle has a role in inducing immunity able to prevent oocyst shedding in cats (Dubey, 2017). Identification of the initial steps necessary for induction of protective immunity against oocyst shedding is necessary to move forward improved vaccination strategies.

Contrary to tachyzoites, bradyzoites are not easily manipulated *in vitro* and developing alternative systems to animal model is becoming a major issue in biology. *In vitro* differentiation models using stress conditions applied to tachyzoites have been demonstrated to induce cyst formation. These include alkaline stress, heat shock treatment, cytokine (IFN-γ), nutrient deprivation, and chemical/drug treatments. The most commonly used method is alkaline treatment and mostly fibroblasts and macrophages are used as host cells. The spontaneous *in vitro* differentiation of bradyzoites in the absence of extrinsic stress, offer new perspectives to develop more efficient differentiation models (Cerutti et al., 2020). Indeed, Paredes-Santos et al. (2016) demonstrated the ability of EGS tachyzoite strain to convert in intracellular cysts/bradyzoites, in different cell types and under physiological pH with a thick wall such as that of mature cysts isolated from mice. Moreover, this provides interesting perspectives to vaccinate livestock animals with bradyzoites through the oral route to induce efficient mucosal immune responses to tackle the parasite at its portal of entry. Additionally, recent advances in understanding the mechanisms behind stage switching provide potentially interesting perspectives for “spontaneous” *in vitro* switch from tachyzoite to bradyzoite stage. For example, parasites (type II or I) in which PKAc3, a protein kinase responsible for cAMP dependent tachyzoite maintenance while suppressing differentiation to bradyzoites, is deleted, have high rates of bradyzoite formation (CST1cyst wall positive vacuoles) (Sugi et al., 2016). Deletion of the transcription factor AP2IV-4 that has been shown to directly silence bradyzoite mRNA and protein expression in tachyzoite, improved also the *in vitro* switch from tachyzoite to bradyzoite stage (Radke et al., 2018). Bradyzoites generated in these models have not yet been fully characterized. They may be more heterogeneous and not equivalent to *in vivo* generated bradyzoites. We do not know if they are infectious by the oral route and able to initiate the enteric parasite cycle.

Until now, only recombinant attenuated tachyzoites were shown to be able to provide protection following challenge against chronic toxoplasmosis. Whether or not bradyzoites would be more efficient than tachyzoites in reducing brain cysts loads has not been evaluated. The ability of bradyzoites to induce a bradyzoite specific immune response following oral infection with cysts remains controversial. In mice orally infected with *T. gondii* cysts, IgG directed against specific bradyzoite surface antigens SRS9, SAG2C, SAG2X, and SAG2Y were not detected (Kim and Boothroyd, 2005). However, BAG1, a bradyzoite antigen and MAG1 a matrix antigen, were shown to induce an early humoral and cell-mediated immune responses upon human infection (Di Cristina et al., 2004). In order to study the potential role of stage-specific expression and compartmentalization of antigens in the induction of a *T. gondii*-specific CD8^+^ T cell response, a *T. gondii* strain engineering to express a secreted or cytoplasmic β-galactosidase either at the bradyzoite or tachyzoite stage was constructed. The frequencies and kinetics of β-galalactosidase-specific CD8^+^ T cells in infected mice was monitored by MHC class I tetramer staining. Upon oral infection with *T. gondii* cysts, only β-galactosidase-secreting tachyzoites, but not bradyzoites secreting β-galactosidase, induced β-galactosidase-specific CD8^+^ T cell in both the spleen and the brain of infected mice (Kwok et al., 2003). These results suggest that bradyzoites are not able to induce a specific CD8^+^ T cell response at the systemic level. In another study to explore the role of stage-specific expression, parasites (Type II PRU strain) were engineering to express a specific bradyzoite surface protein (SRS9) at both tachyzoite and bradyzoite stages (or a specific tachyzoite surface protein (SAG1) at both tachyzoite and bradyzoite stages). In mice infected intraperitoneally with tachyzoites, expression of SRS9 at both tachyzoite and bradyzoite elicited a specific SRS9 humoral and cellular T cell response that was absent in wild type infection and resulted in reduced cyst burdens in brains of parasite-infected mice (Kim and Boothroyd, 2005). Both the amount and the systemic dissemination of tachyzoites may account for an efficient presentation of SRS9 by tachyzoites to the immune system (in the spleen and also in the brain). More recently, by the use of parasites expressing the CMHI peptide of OVA in fusion with the GRA6 protein at the tachyzoite stage or at both tachyzoite and bradyzoite stages, comparable OVA specific CD8^+^ T cell responses were elicited when expressed only by tachyzoite or by both stages in the spleen and the brain of mice infected intraperitoneally with tachyzoites. Compared to the parental strain, both parasites expressing GRA6-OVA peptide at tachyzoite stage or at tachyzoite and bradyzoite stages reduced cyst burdens in the brains (Salvioni et al., 2019). These studies suggest that both the amount and the systemic dissemination of tachyzoites may account for an efficient presentation of bradyzoite specific or antigens shared by both tachyzoites and bradyzoites, by tachyzoites to the immune system (in the spleen and also in the brain). On the contrary, specific bradyzoite antigens seem poorly immunogenic due to the location and quiescent metabolic activity of bradyzoites along with the timing of the immune response that is able to see them. Thus, a tachyzoite expressing multiple bradyzoite antigens could be a good candidate vaccine to induce efficient immune responses against both tachyzoite and bradyzoite stages. Indeed, intraperitoneal inoculation of tachyzoites expressing cyst wall proteins (e.g. BPK1, MCP4, CST1) and the bradyzoite surface antigen SRS9, by deleting the transcription factor AP2IV-4 that has been shown to directly silence bradyzoite mRNA and protein expression in tachyzoite, were unable to form cysts in brain tissue and a potent immune response characterized by increases inflammatory monocytes, IFN-γ and higher numbers of both CD8^+^and CD4^+^ T-cells was observed (Radke et al., 2018). These results suggest an increased parasite control by the mice compared to the parent strain. However, protection against an oral challenge with *T. gondii* cysts has not been performed in this study.

For pigs especially in farms allowing outdoor access and sheep, infections occur after ingestion of *T. gondii* oocysts shed by cats in the environment. Vaccination through the oral route with oocysts/sporozoites would induce efficient mucosal immune responses to tackle the parasite at its portal of entry. The immune responses induced by oocysts at both mucosal and systemic levels remain poorly understood in livestock animals and even in mice. In humans and pigs, oral infection with *T. gondii* oocysts induced a systemic production of antibodies (IgG – IgM) directed against specific sporozoite/oocyst antigens (Hill et al., 2011; Santana et al., 2015; Liu et al., 2019). Salivary IgA against the sporozoite antigen TgERP were also detected in *T. gondii* infected human (Mangiavacchi et al., 2016). Moreover, in mice orally infected with *T. gondii* oocysts, serum IgG antibodies directed against the sporozoite specific CCp5A protein were detected (Santana et al., 2015). These observed humoral responses suggest that reactions involving sporozoite antigen specific B cells and follicular CD4^+^ T cell can take place in germinal centers. The lack of knowledge, is due at least in part to technical difficulties to obtain purified oocysts. To date, oocysts are obtained from infected cats. Until recently, the molecular determinants that identify cats as the definitive host for sexual development were unknown. Martorelli Di-Genova et al. (2019) found that inhibition of murine delta-6-desaturase, an enzyme for the conversion of linoleic acid to arachidonic acid and supplementation of their diet with linoleic acid allowed *T. gondii* sexual development in mice. Mouse derived oocysts sporulate and are infectious. They also obtained sexual development in cell culture mouse cells supplemented with both linoleic acid and SC-26196 a specific inhibitor of the delta-6-desaturase enzyme, approximately 26% of the *T*. *gondii* vacuoles expressed both BRP1 and GRA11B. More recently, the *T. gondii* microrchidia (MORC) protein was identified as an upstream transcriptional repressor of sexual commitment (Farhat et al., 2020). Deletion of the MORC protein resulted in the induction *in vitro* of the expression of cyst wall and sporozoite proteins. These works open new perspectives for alternative *in vitro* production of sexual *T. gondii* stages and these new advances provide potentially interesting new perspectives for a better understanding of this life cycle stage, stage-host interactions and host immune responses.

If vaccination with sexual stages is not achievable, an alternative could be tachyzoites that express proteins, fragment of proteins, or chimeric proteins carrying T and B cell epitopes of antigens expressed by bradyzoite/sporozoite, to get homogeneous and reproducible parasite populations carrying these epitopes (**Figure 1**). The subcellular localization of these epitopes would be important to get the most immunogenic context. Using OVA as a model antigen, it has been shown that tachyzoites expressing secreted OVA in the vacuole but not those that express a cytosolic OVA stimulated OVA specific CD4^+^ T cells (Pepper et al., 2004). In addition, tachyzoites expressing secreted OVA in the vacuole, and to a less degree those that express a surface membrane bound OVA stimulated OVA specific CD8^+^ T cells. But tachyzoites expressing intracellular OVA (either in the cytosol, the mitochondrion or inner membrane complex) were unable to stimulate OVA specific CD8^+^ T cells (Gregg et al., 2011). Thus, both specific CD4^+^ and CD8^+^ T cell responses would be promoted when the protein is secreted. Furthermore the fate of the secreted protein may also influence the CD8^+^ T cell response. For example studies using GRA6, a *T. gondii* dominant antigen, showed that GRA6 MHC I presentation is favored when the protein is inserted in the PVM and when its C-terminal MHC I epitope is exposed to the host cell cytosol (Lopez et al., 2015; Buaillon et al., 2017). To increase MHC I presentation, strategies facilitating perturbation/disruption of the PVM to release PV localized antigens in the host cell cytosol may be developed. In previous studies, IRGs proteins were shown to enhance antigen presentation of *T. gondii* antigens in mouse embryonic fibroblasts (Lee et al., 2015). Recently, deletion of ROP5, ROP18 or GRA7, all implicated in resistance to IRGs that mediate parasite killing in mice, was shown to increase PV clearance and presentation of soluble PV antigen by MHC I molecules (Rommereim et al., 2019).

Another property of the recombinant live vaccine candidate would be the absence of persistent parasites in the vaccinated host. A growing list of bradyzoite or cyst wall specific gene knockouts, in attempts to block parasite conversion, can be found in the literature. The vast majority of knockouts resulted in reduced cyst burdens in chronically infected mice. For few of them, brain cysts were not detected. Loss of the Ca^2+^-dependent protein kinase CDPK2 results in the hyperaccumulation of amylopectin in bradyzoites, leading to parasite death and abrogation of *in vivo* cyst formation (Uboldi et al., 2015). However, CDPK2 is not a major determinant of tachyzoite virulence, as CDPK2-deficient parasites exhibited no defect in acute virulence. GRA17 mediates the tachyzoite PVM permeability to nutrients, loss of GRA17 results in a failure of GRA17 deficient parasites to reach the brain and form cysts (brain cysts and *T. gondii* DNA were not detected). The viability of bradyzoites obtained from *in vitro* GRA17-deficient parasites was significantly reduced compared to wild-type bradyzoites. Furthermore, GRA17-deficient parasites were avirulent in mice (Paredes-Santos et al., 2019). Loss of the cyst wall GRA protein CST2 results in virulence defect and brain cysts were not detectable in mice with chronic infection (Tu et al., 2019). However, parasite load was not further investigated by PCR and CST2-deficient parasites are still able to form *in vitro* cysts. Recently, Waldman et al. (2020) identify a Myb-like transcription factor (BFD1) necessary for differentiation of tachyzoite to bradyzoite in cell culture and in mice. BFD1-deficient *T. gondii* tachyzoites are unable to form cysts in mice, thus BFD1-deficient *T. gondii* tachyzoites could serve as a basis for developing live vaccine strain, capable of proliferating robustly yet unable to enter a chronic state.

Finally to improve the immunogenicity of a live vaccine, whatever the *T. gondii* stage, deletion of some genes used by the parasite to manipulate the host cell and/or overexpression of some genes that may potentiate the innate immune response may be achieved. For example, deletion of TgIST in a type II strain, a secreted effector conserved across strains, that has been shown to inhibit both STAT1 and STAT1/STAT2-mediated transcriptions and to block induction of type II and type I IFN- activated genes, results in increased parasite clearance in IFN-γ-activated cells and reduced mouse virulence (Gay et al., 2016; Olias et al., 2016; Matta et al., 2019). It has been shown that GRA15 from the type II strain activates NF-kB pathway while GRA15 from the type I does not. Expression of GRA15 type II in a type I strain successfully activated the signaling pathways of NF-kB and induced expression of CD40 on infected mouse macrophages and human THP-1 differentiated macrophages (Morgado et al., 2014).

Furthermore, as the primary infection with a specific strain does not confer complete protection against other *Toxoplasma* strains in both animal and human hosts (Jensen et al., 2015; Costa et al., 2018; Zulpo et al., 2018), the choice of the strain for a live vaccine will be dependent on its ability to induced heterologous immune response and could be adapted to its geographical use. If in Europe and North America, the *T. gondii* strains clonal lineages (types II, III and 12) predominate, non-clonal genotypes are abundant and more genetically diverse in South and Central America (Galal et al., 2019).

### Humans

#### Human Vaccine Development

Numerous vaccine trials have been conducted in mice yielding important insights into toxoplasmosis vaccine development. However, predictive protectivity estimated in mice may be not extrapolated to human, considering the differences in immune responses between mouse and human. Sheep and pig may be more relevant animal models than the mouse to test potential human vaccines. Both sheep and pig have clinical similarities responses to *T. gondii* compared to human (Nau et al., 2017; Betancourt et al., 2019). In particular, pigs like humans are exposed to both *T. gondii* cysts and oocysts (Stelzer et al., 2019). In addition, the physiology and the immune system of pigs are closely that of humans (Meurens et al., 2012). A key difference between mice and humans, sheep and pigs, is the innate immune response. In mice, TLR11 and TLR12 recognition of *T. gondii* profilin induced IL-12 production. Humans, pigs and sheep do not have functional equivalents to murine TLR11 and TLR12. In addition, humans lack most IRGs, contrary to mice where the IFN-γ inducible IRGs proteins play an essential role in IFN-γ-mediated immunity to *T. gondii*. Another species-specific immune defense mechanism involves granulysin. Granulysin (GNLY), an antimicrobial peptide contained in cytotoxic granules in human natural killer cells and cytolytic T lymphocytes but not in mouse cells, has been shown to mediate *T. gondii* death *in vitro* and GNLY-transgenic mice are protected against infection by *T. gondii*, and survive infection that are lethal to wild-type mice (Dotiwala et al., 2016).

To stimulate protective cell mediated immune responses to *T. gondii*, in particular a CD8^+^ T cell protective immune response, live vaccines are likely effective for generating such a response. Furthermore, live vaccines did not need adjuvant as immunity booster and they give the opportunity to immunize with all the diversity and complexity of *T. gondii* antigens. However, live vaccines would require passing considerable regulatory hurdles that may impede their use in human. One possibility to immunize with all the diversity and complexity of *T. gondii* antigens is the use of *T. gondii* extract. The second one is the use of delivery systems and adjuvants that improved antigen bioavailability and elicited cellular immune responses, especially Th1 type (**Figure 1**). Such an approach has been applied to sheep. Intranasal immunization with *T. gondii* tachyzoite antigens (crude extract) encapsulated into poly(d,l-lactide-co-glycolide) (PLG) micro and nanoparticles plus cholera toxin, induced both systemic and mucosal humoral responses, and cell-mediated responses. However, only a slight modification of the febrile response to oral challenge with sporulated *T. gondii* oocyst was observed in animals immunized with particulate *T. gondii* antigens and cholera toxin compared to control group (Stanley et al., 2004). Intranasal vaccination of sheep with a total extract of *T. gondii* tachyzoite proteins associated with maltodextrin nanoparticles (DGNP), generated specific Th1-cellular immune responses and led to a marked decrease of brain cysts compared with the non-vaccinated group after oral challenge with sporulated *T. gondii* oocysts (Ducournau et al., 2020). To our knowledge, *T. gondii* extracts from *T. gondii* bradyzoite or sporozoite stages have not been investigated in sheep and pigs. The technical challenges to obtain such extracts in large quantities have hindered their use in vaccination trials on large animals.

Despite significant differences between mice and humans, HLA transgenic mice may be considered as good approximation of human responses. To develop peptide-based vaccines created with identified endogenous *T. gondii*–derived epitopes recognized by human CD8^+^ T cells, HLA A02, A03, and B07 transgenic mice have been use. These HLA molecules are present in more than 90% of the human population. The selected peptides, derived from tachyzoites, bradyzoites or sporozoites antigens administrated subcutaneously with a universal helper T cell epitope, PADRE, and adjuvants (GLA-SE combined or not with Pam2Cys, to adjuvant MHC Class I restricted CD8^+^ T cells) elicited IFN-γ and reduced parasite burden in HLA A02, A03, and B07 transgenic mice following intraperitoneal challenge with type II tachyzoites that express firely luciferase for *in vivo* detection (Cong et al., 2010; Tan et al., 2010; Cong et al., 2011; Cong et al., 2012; El Bissati et al., 2016). To further increase the immunogenicity of the peptides, multi-epitopes vaccines instead of pool peptides have been constructed by El Bissati et al. (2017). They included 5 HLA A03 restricted epitopes (from SAG1, SAG2C, SRS52A, GRA5 and GRA6), a universal CD4^+^ T cell epitope PADRE, all arranged to optimize proteasomal cleavage and flagellin as a scaffold and TLR5 agonist to create Self-Assembling Protein Nanoparticles (SAPNs). These SAPNs known to improve the immune response due to the repetitive display of antigens were also adjuvanted with TLR4 ligand emulsion adjuvant (GLA-SE). With this formulation, CD8^+^ T cells were activated and a significant brain cyst reduction in A03 transgenic mice was observed following intraperitoneal challenge with tachyzoites that express firely luciferase (El Bissati et al., 2017).

#### Vaccination Perspectives for Humans

Until now only few studies have addressed the identification of epitopes derived from *T. gondii* proteins presented by HLA major histocompatibility complex molecules I and relatively few *T. gondii* protective epitopes have been identified to date. As *T. gondii* has a large genome (around 65 Mb), screening with synthetic peptides spanning all the proteins encoded in the genome with CD8^+^ T cells from immune individuals is not applicable. As experiments using parasites engineered to express model antigen have previously shown that transgenic antigen-specific CD8^+^ T cells are detected in transgenic parasite-infected mice only if the transgenic antigen is expressed as a secreted/membrane bound protein, a set of secreted/membrane bound proteins were selected and bioinformatics algorithms have been used to identify MHC binding peptides. In these studies, a protective immunity was obtained in transgenic HLA mice that could be improved with additional CD8^+^ T cell peptide candidates from the different stages of *T. gondii*, and also by the addition of CD4^+^ T cell-eliciting epitopes. The immunogenicity of predicted *T. gondii* CD4^+^ epitopes in transgenic mice has not been evaluated to date. Transgenic mice expressing both MHC class I and MHC class II molecules from human origin could be useful in epitope screening and vaccine trials. For example, HLA-A02.1-/HLA-DR1-transgenic H-2 class I-/class II-knockout mice have been used in DNA vaccination trials with a hepatitis B antigen and proved to be efficient in inducing a protective cellular and humoral response. This response was more efficient than the responses induced in transgenic mice expressing either HLA-0A2 or HLA-DR1 alone (Pajot et al., 2004). An immunosense vaccine for humans encompassing CD8, CD4, and B cell epitopes from different stages of *T. gondii* and tested in transgenic mice expressing both MHC class I and II from human origin will be interesting to look at although engineering these mice with different variant HLAs will be challenging and hard to manage. Furthermore, the pathways by which parasite antigens may be acquired and processed for MHC presentation are multiple, depending notably on the location, trafficking of the protein within parasites and host cells (Jensen, 2016; Tsitsiklis et al., 2019). Further studies are needed to understand the detailed mechanisms of these pathways, from entry to early dissemination with cysts or oocysts at mucosal sites. Until now, most studies focused on tachyzoite infected cells.

Protection against *T. gondii* mainly depends on a cellular-mediated immune response, nonetheless antibodies may contribute to protection. They can act on the extracellular tachyzoites by opsonization, complement fixing, neutralization/inhibition of host cell invasion. Those directed against antigens that are implicated in attachment to, or penetration in the host cells of the different *T. gondii* stages are of particular interest. Numerous linear B cell epitopes have been identified to date and are useful for *T. gondii* serological diagnosis (Maksimov et al., 2012; Arranz-Solís et al., 2019). Noteworthy, most of naturally recognized epitopes are discontinuous and are more challenging to identify. Analysis of the three-dimensional (3D) X-ray structure of an antigen by using bioinformatical software programs is needed for the search of these conformational epitopes.

Studies aiming at the identification of CD8^+^, CD4^+^, and B cell specific *T. gondii* epitopes are still needed to rationally design an efficient multi-epitope vaccine engineered to focus both T and B cells responses toward a set of critical epitopes selected from a wide range of antigens in order to diversify the scope of the induced immune responses. In parallel, knowledge of new adjuvants and vaccine delivery systems will be useful in the development of effective vaccines.

## Conclusions

Recent advances in deciphering the *T. gondii* life stage conversions including those throughout the sexual cycle, pave the way for *in vitro* studies to tackle the biological questions related to *T. gondii* and will hopefully open new perspectives to move toward effective vaccines. As *T. gondii* biology is intimately linked to the immune response, identification of the initial steps necessary for induction of protective immunity in the relevant host is also critical. Intestinal organoid models that are able to reproduce the *in vivo* situation, offer the opportunity to provide insights into these early host parasite interactions (Betancourt et al., 2019). New tools aiming to identify protein composition of the different *T. gondii* stages including oocyst and cyst walls and stage conversions such as interactome constructions using proteins identified through BioID or RNA single cell sequencing (Xue et al., 2020) will help to better understand the parasite biology and to identify possible new vaccine candidates. In addition, recently, a comprehensive genomescale metabolic model (GEM) of the *T. gondii* tachyzoite metabolic network that incorporates genetic, transcriptomic, and metabolomic data has been developed (Krishnan et al., 2020), should be applied to *T. gondii* bradyzoite or sexual stages to decrypt/to decipher metabolic cues and molecular signaling cascades that trigger bradyzoite transformation and switching to the sexual stages. Our progress toward understanding the biology of *T. gondii* and new tools and technologies enable new opportunities in vaccine development.

## Author Contributions

M-NM and ID-P conceptualized the content and wrote the manuscript. ZL made changes and clarified the document. M-NM generated the tables and ZL generated the figure with the aid of BioRender (©biorender.com.). All authors contributed to the article and approved the submitted version.

## Funding

Funding for this study was provided by the National Research Institute for Agriculture, Food and Environment and the University of Tours.

## Conflict of Interest

The authors declare that the research was conducted in the absence of any commercial or financial relationships that could be construed as a potential conflict of interest.
